# Combining untargeted and targeted metabolomic profiling reveals principal differences between osteopenia, Osteoporosis and healthy controls

**DOI:** 10.1007/s40520-024-02923-3

**Published:** 2025-01-21

**Authors:** Bing Tan, Yan Cheng, Junfeng Li, Yuhao Zheng, Cong Xiao, Haoning Guo, Bing Wang, Jianyuan Ouyang, Wenmin Wang, Jisheng Wang

**Affiliations:** 1 The Third Hospital of Mianyang, Sichuan Mental Health Center, Mianyang, 621000 China; 2 The Yangtze River Delta Biological Medicine Research and Development Center of Zhejiang Province, Yangtze Delta Region Institution of Tsinghua University, Hangzhou, Zhejiang 314006 China

**Keywords:** Osteoporosis, Osteopenia, Postmenopausal women, Metabolomics, Biomarker

## Abstract

**Background:**

Osteopenia (ON) and osteoporosis (OP) are highly prevalent among postmenopausal women and poses a challenge for early diagnosis. Therefore, identifying reliable biomarkers for early prediction using metabolomics is critically important.

**Methods:**

Initially, non-targeted metabolomics was employed to identify differential metabolites in plasma samples from cohort 1, which included healthy controls (HC, *n* = 23), osteonecrosis (ON, *n* = 36), and osteoporosis (OP, *n* = 37). Subsequently, we performed targeted metabolomic validation of 37 amino acids and their derivatives in plasma samples from cohort 2, consisting of healthy controls (HC, *n* = 10), osteonecrosis (ON, *n* = 10), and osteoporosis (OP, *n* = 10).

**Results:**

The non-targeted metabolomic analysis revealed an increase in differential metabolites with the progression of the disease, showing abnormalities in lipid and organic acid metabolism in ON and OP patients. Several substances were found to correlate positively or negatively with bone mineral density (BMD), for example, N-undecanoylglycine, sphingomyelins, and phosphatidylinositols exhibited positive correlations with BMD, while acetic acid, phenylalanine, taurine, inosine, and pyruvic acid showed negative correlations with BMD. Subsequently, targeted validation of 37 amino acids and their metabolites revealed six amino acids related to ON and OP.

**Conclusion:**

Significant metabolomic features were identified between HC and patients with ON/OP, with multiple metabolites correlating positively or negatively with BMD. Integrating both targeted and non-targeted metabolomic results suggests that lipid, organic acid, and amino acid metabolism may represent important metabolomic characteristics of patients with OP, offering new insights into the development of metabolomic applications in OP.

**Supplementary Information:**

The online version contains supplementary material available at 10.1007/s40520-024-02923-3.

## Background

Osteoporosis (OP) is a prevalent, chronic, systemic skeletal disorder characterized by diminished bone mass and density, alongside disruption in bone tissue microarchitecture. These changes result in compromised bone strength and an elevated fracture risk [1, 2]. Globally, OP affects over 200 million individuals, predominantly postmenopausal women [3]. The disease’s progression, linked to decreased estrogen levels, involves enhanced bone resorption and impeded bone formation, disrupting bone remodeling [4]. The National Health Commission of the People’s Republic of China’s epidemiological survey indicates a significant prevalence of OP in Chinese individuals over 50, with rates of 32.1% in women and 6% in men [5]. OP’s primary complication, fractures, not only increases mortality and disability but also imposes significant economic burdens. In 2000, the direct fracture-related costs in the European Union were estimated at 32 billion euros, projected to double by 2050 [3].

Osteopenia (ON) represents a precursor state to OP, marked by reduced bone mineral density (BMD) not severe enough to be classified as OP. The key distinction between ON and OP lies in the degree of BMD reduction and fracture risk [6]. Dual-energy x-ray absorptiometry (DXA) remains the clinical gold standard for BMD measurement and ON/OP diagnosis [7]. A T-score between − 1.0 and − 2.5 indicates ON, whereas a score below − 2.5 confirms OP [8]. However, ON/OP often develops asymptomatically, eluding early detection until fracture occurrence [9]. Thus, early diagnosis and intervention are essential for mitigating disease progression. Identifying biomarkers for high OP risk is vital for enhancing patient outcomes and reducing healthcare burdens. Established OP risk factors include BMD, gender, age, smoking, alcohol use, and metabolic disorders like diabetes [10, 11]. Although fracture prediction tools incorporating these factors exist, their predictive accuracy remains suboptimal [12]. Given OP’s multifactorial etiology, accurate prediction poses a considerable challenge.

Metabolomics, a field focusing on the comprehensive analysis of metabolites within biological systems, employs chromatography-mass spectrometry techniques to study metabolite composition and fluctuations [13]. Metabolites, as end products of metabolic pathways, offer insights into an organism’s metabolic state and are instrumental in identifying disease-related metabolic abnormalities. Metabolomics has been utilized in predicting various diseases, including metabolic, vascular, respiratory disorders, and cancers. A notable example is a study using the UK Biobank data, which developed a deep residual multitask neural network for blood metabolome analysis. This network effectively discerns the metabolomic profiles of 24 common diseases [14]. Recent research has increasingly applied metabolomics to OP and ON, identifying distinct metabolic variations in affected individuals [15, 16]. These findings underscore metabolomics’ potential in early OP detection, personalized medicine, and new therapeutic target identification. However, disparities in study results and the prevalence of false positives and negatives necessitate caution. Reliable biomarkers require repeated validation across diverse populations for higher trustworthiness.

This study aims to explore the metabolomic differences between ON/OP patients and healthy controls through untargeted metabolomics, in order to identify potential biomarkers. By integrating reports from relevant literature, potential biomarkers will be subject to targeted validation using an independent validation set, enhancing the robustness and reliability of these biomarkers across diverse populations.

## Materials and methods

### Study population

The study utilized plasma samples from postmenopausal women at the Third People’s Hospital of Mianyang City. It comprised two cohorts: cohort 1 and cohort 2. Participants in both cohorts were categorized into three groups based on their BMD T-scores: those with a T-score above − 1.0 were classified as the healthy control group, those with T-scores between − 1.0 and − 2.5 as the ON group, and those with T-scores below − 2.5 as the OP group. Cohort 1 consisted of 96 participants (HC = 23, ON = 36, OP = 37), while cohort 2 had 30 participants (HC = 10, ON = 10, OP = 10). All participants were voluntary Chinese residents at the Third People’s Hospital of Mianyang City. The study received approval from the Ethics Committee of the Third People’s Hospital of Mianyang City (2024, Reviewed, No.3), with all participants providing informed consent.

Inclusion criteria: (1) Postmenopausal women; (2) Underwent BMD measurement at the time of visit. Exclusion criteria: (1) Presence of conditions potentially causing osteoporosis, such as thyroid or parathyroid disorders, chronic kidney or liver disease, stroke, coronary heart disease, malignancies, hematologic disorders, tuberculosis, severe malnutrition, other chronic wasting diseases, or autoimmune diseases. (2) Usage of medications affecting BMD within the past three months, including cortisone, sex hormones, levothyroxine, immunosuppressants, antiepileptic drugs, etc. (3) Incomplete study data. (4) History of conditions or surgeries leading to amenorrhea, like polycystic ovary syndrome (PCOS), or history of ovarian or uterine surgery.

### Untargeted metabolomic profiling

Blood samples were collected in vacuum tubes containing anticoagulants and centrifuged at 3000 rpm for 10 min. The supernatant (plasma) was then carefully transferred to clean centrifuge tubes, quickly frozen in liquid nitrogen for 15 min, and subsequently stored at -80 °C.

Upon thawing on ice, metabolites were extracted using pre-cooled 80% methanol buffer. Quality control (QC) samples, created by pooling extracts from all metabolites in Cohort 1, were analyzed after every ten test samples to ensure liquid chromatography-mass spectrometry (LC-MS) system stability throughout the collection process. Separation and identification of samples were executed using LC-MS. The column temperature was maintained at 40 °C, with elution utilizing solvent A (5 mM ammonium acetate and 5 mM acetic acid) and solvent B (Acetonitrile) according to a specific gradient program. Chromatographic separations were conducted with an UltiMate 3000 UPLC System (Thermo Fisher Scientific, Bremen, Germany) and an ACQUITY UPLC T3 column (100 mm*2.1 mm, 1.8 μm, Waters, Milford, USA).

Metabolites were detected using a Q-Exactive high-resolution tandem mass spectrometer (Thermo Scientific), with precursor spectra acquired from 70 to 1050 m/z at a resolution of 70,000 and an AGC target of 3e6. Raw mass spectrometry data were converted into mzXML format using MSConvert software, followed by peak extraction with XCMS software. Metabolite annotation employed databases like HMDB and KEGG, and differential metabolites were quantified and screened using metaX software.

### Targeted metabolomic profiling

The protocol for sample collection and metabolite extraction in targeted metabolomics paralleled that of non-targeted metabolomics. The targeted metabolomics analysis focused on plasma samples derived from cohort 2. QC samples, created by pooling extracts from all metabolites in cohort 2. The separation and identification of metabolites were facilitated through the use of LC-MS, which enabled the precise quantification of 37 amino acids. The mobile phase A was composed of a 10 mmol/L ammonium formate aqueous solution, containing 0.1% formic acid, whereas mobile phase B was a 10 mmol/L ammonium formate solution in methanol-water (volume ratio = 9:1), also with 0.1% formic acid. Qualitative analysis was achieved by comparing the retention times and Multiple Reaction Monitoring (MRM) fragment ions against standard reference materials. Quantitative analysis was performed using external standards. Samples that exhibited concentrations beyond the calibration curve range were diluted appropriately and reanalyzed, with the resultant data from the dilution process being utilized for final quantification.

### Statistical analysis

All data were processed using IBM SPSS Statistics 26 and were presented as mean ± standard deviation (mean ± SD) and numerical values and percentages (n, %). T test or Mann-Whitney U test was used to compare the two groups. One-way analysis of variance (ANOVA) was used to compare the three groups. *P* < 0.05 was considered statistically significant. Performing receiver operating characteristic (ROC) analysis to assess the discriminatory power of each differential metabolite.

## Results

### Study design and participant cohorts

**Fig. 1:**
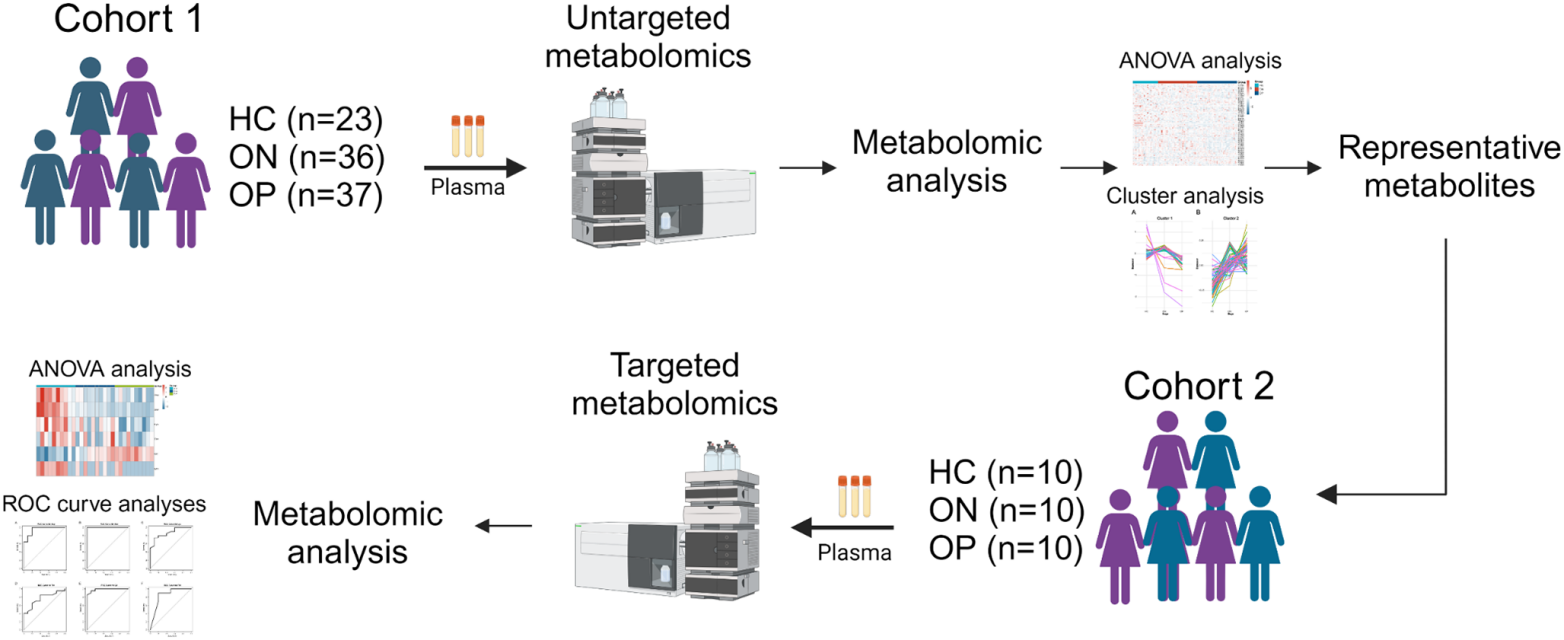
Overview of the study design. The illustration was created with BioRender

Our study aimed to explore the differential metabolites among the healthy controls (HC), ON, and OP patients. Initially, plasma samples from cohort 1 were subjected to non-targeted metabolomics analysis to identify differential metabolites. Subsequently, plasma from cohort 2 was analyzed for targeted validation of these metabolites, seeking potential biomarkers for ON/OP. The design of this study is illustrated in Fig. 1. Tables 1 and 2 present clinical characteristics and baseline data for patients in cohort 1 and cohort 2, respectively. In cohort 1, compared to the HC group, both the ON and OP groups exhibited lower t-scores, with no significant differences observed in other characteristics. In cohort 2, besides showing lower t-scores, both the ON and OP groups also had significantly higher ages compared to the HC group.

**Table 1:** Clinical characteristics and demographics of cohort 1

Characteristics	HC	ON	OP
Sample size n (%)	23 (23.96%)	36 (37.50%)	37 (38.54%)
Age (year)	61.26 ± 6.36	61.19 ± 5.90	63.35 ± 6.93
BMI (kg/m^2^)	24.24 ± 3.32	23.67 ± 3.68	22.89 ± 2.70
T-score	0.12 ± 0.47	-1.36 ± 0.35^*^	-3.26 ± 0.66^*#^
FBG (mmol/L)	5.52 ± 0.65	5.61 ± 0.77	5.65 ± 0.94
Cholesterol (mmol/L)	4.67 ± 0.59	4.54 ± 0.66	4.52 ± 0.92
TG (mmol/L)	1.38 ± 0.70	1.25 ± 0.42	1.22 ± 0.42
HDL (mmol/L)	1.48 ± 0.31	1.46 ± 0.33	1.69 ± 0.64
LDL (mmol/L)	2.99 ± 0.56	2.87 ± 0.67	2.68 ± 0.76

Notes BMI, body mass index; FBG, fasting blood glucose; TG, triglycerides; HDL, high-density lipoprotein; LDL; low-density lipoprotein. * p-value < 0.05 vs. HC group; # p-value < 0.05 vs. ON group. All data were presented as (mean ± SD) and (n, %)

**Table 2:** Clinical characteristics and demographics of cohort 2

Characteristics	HC	ON	OP
Sample size n (%)	10 (33.33%)	10 (33.33%)	110 (33.33%)
Age (year)	54.40 ± 6.06	60.40 ± 5.97^*^	66.70 ± 3.20^*#^
BMI (kg/m^2^)	22.88 ± 2.48	23.28 ± 1.63	22.18 ± 2.47
T-score	0.55 ± 0.58	-1.60 ± 0.31^*^	-3.77 ± 0.54^*#^
FBG (mmol/L)	4.66 ± 0.44	5.58 ± 1.96	6.11 ± 2.35
Cholesterol (mmol/L)	4.65 ± 0.97	4.77 ± 0.58	4.91 ± 0.60
TG (mmol/L)	0.98 ± 0.32	1.27 ± 0.35	1.24 ± 0.30
HDL (mmol/L)	1.59 ± 0.39	1.64 ± 0.36	1.48 ± 0.34
LDL (mmol/L)	2.92 ± 0.86	2.92 ± 0.49	3.19 ± 0.45

Notes BMI, body mass index; FBG, fasting blood glucose; TG, triglycerides; HDL, high-density lipoprotein; LDL; low-density lipoprotein. ^*^ p-value < 0.05 vs. HC group; ^#^ p-value < 0.05 vs. ON group. All data were presented as (mean ± SD) and (n, %)

### Untargeted metabolomic profiling

#### Metabolite Identification

Through untargeted metabolomics analysis, we identified a total of 1242 metabolic features from 96 plasma samples in cohort 1, with 506 in positive ion mode and 736 in negative ion mode. The vast majority of the metabolites identified were Lipids and lipid-like molecules (such as glycerophospholipids, fatty acyls, sphingolipids, prenol lipids, and sterol lipids). Other major metabolite superclass included organic acids and derivatives, benzenoids, etc. A small portion were Purine nucleosides, such as inosine and methyladenosine. Detailed information on metabolic features is presented in Supplementary Table 1.

### Statistical analysis of 96 plasma samples in untargeted metabolomics

**Fig. 2:**
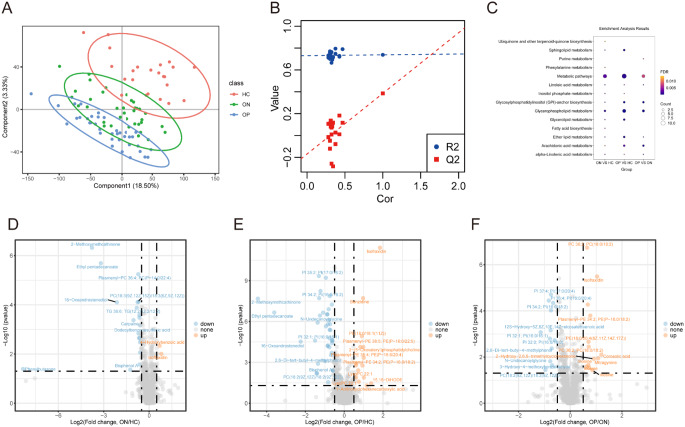
Partial least squares discriminant analysis and statistical analysis of differential metabolites. (**A**) PLS-DA of the metabolomics data from HC, ON, and OP groups, (**B**) Permutation tests of PLS-DA models, (**C**) Kyoto Encyclopedia of Genes and Genomes (KEGG) metabolic pathways enriched by significantly differential metabolites in ON versus HC, in OP versus HC, and in OP versus ON, (**D**) Volcano plots of the significantly differential metabolites in ON versus HC, (**E**) in OP versus HC, and (**F**) in OP versus ON

Partial least squares discriminant analysis (PLS-DA) was utilized to evaluate intergroup differences. The PLS-DA score plot showed that although there was a certain degree of overlap between HC, ON, and OP groups, they could be clearly divided into three categories (Fig. 2A). It indicated that each set of data maintains relative independence in the principal component space, suggesting that there may be potential biological differences. Moreover, permutation tests confirmed the robustness and non-overfitting of the OPLS-DA model (Q2 < 0), ensuring the accuracy of metabolic differentiation analysis (Fig. 2B).

Subsequently, we performed pairwise comparison analysis of the three groups (HC, ON and OP) to identify differential metabolites. In the ON versus HC comparison, 21 differential metabolites (DMs) were identified, comprising 2 upregulated and 19 downregulated DMs (Fig. 2D and Supplementary Table 2). The OP versus HC comparison revealed 53 DMs, including 15 upregulated and 38 downregulated DMs (Fig. 2E and Supplementary Table 3). As the severity of the disease increased, more DMs were detected. The ON versus OP comparison indicated 25 DMs, with 13 upregulated and 12 downregulated, signifying significant metabolic discrepancies between these groups (Fig. 2F and Supplementary Table 4). N-undecanoylglycine emerged as a significant DM in all three comparisons: ON versus HC, OP versus HC, and ON versus OP. Inosine showed significant downregulation in ON versus OP. It is noteworthy that as the severity of the disease increases, there are disruptions in the metabolism of various lipid substances in the plasma. For example, sphingomyelins (SMs), phosphatidylinositols (PIs), and phosphatidylcholines (PCs) are among them. Furthermore, KEGG pathway analysis revealed that these DMs were predominantly involved in disrupted metabolic processes, including Glycerophospholipid metabolism, Glycerolipid metabolism, and Arachidonic acid metabolism (Fig. 2C).

### The progressive metabolic evolution analysis from HC to OP

**Fig. 3:**
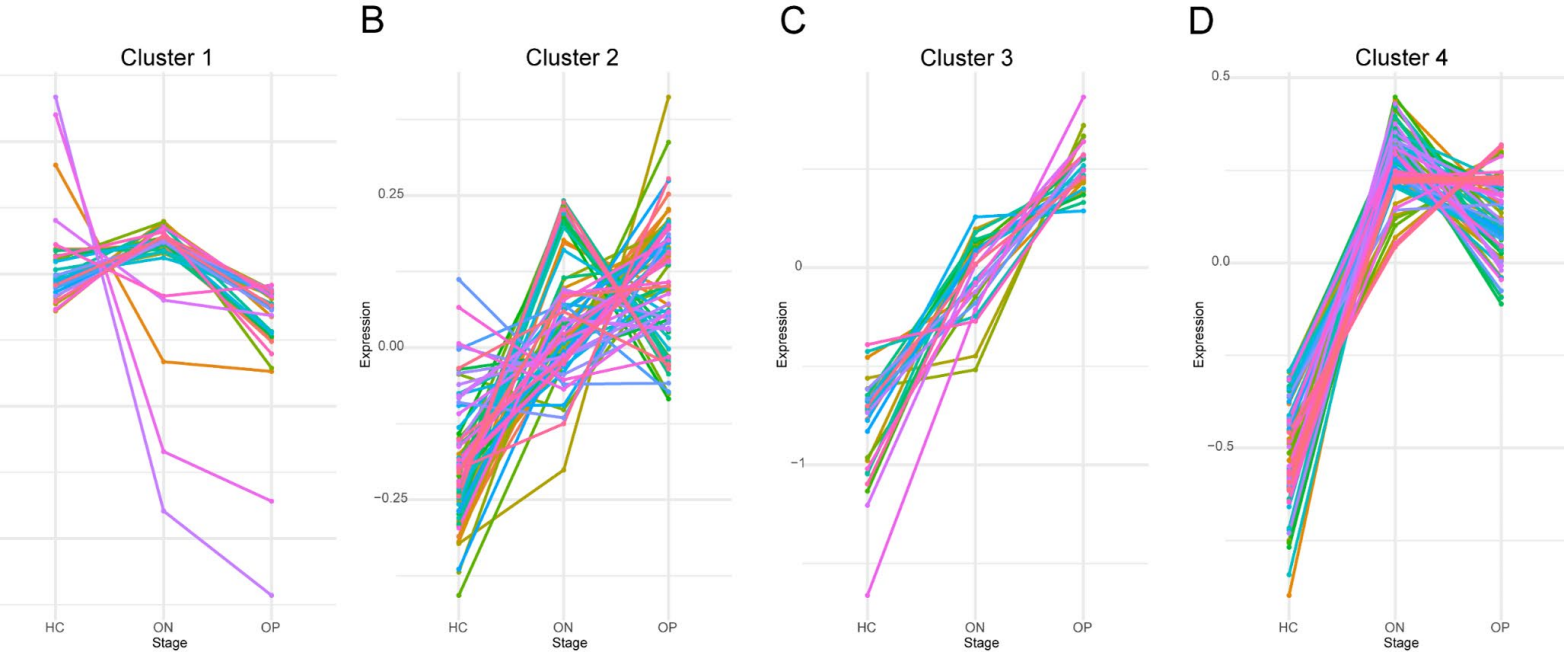
Clustering of metabolic trajectories using differential metabolites among HC, ON, and OP. (**A**) Cluster 1, (**B**) Cluster 2, (**C**) Cluster 3, (**D**) Cluster 4

Utilizing ANOVA analysis of variance, we identified 208 DMs across the HC, ON, and OP groups, as shown in Supplementary Fig. 1. These metabolites span a diverse array of substances, primarily categorized into lipids and lipid-like molecules and organoheterocyclic compounds (Supplementary Fig. 2). To elucidate the metabolic trajectory from HC to OP, c-means clustering was performed on the differential metabolites identified at each stage. This analysis revealed four distinct clusters (Fig. 3A-D), highlighting specific metabolic patterns. Notably, certain substances were significantly enriched in the ON stage, such as uric acid and lysophosphatidic acid, indicating distinct metabolic vulnerabilities that emerge in the early phases of osteoporosis. These could potentially serve as early diagnostic markers and monitoring tools for the disease. Moreover, several substances, including N-undecanoylglycine, SMs, and PIs exhibited a positive correlation with BMD, while acetic acid, phenylalanine, taurine, inosine, and pyruvic acid, exhibited a negative correlation with BMD. Supplementary Table 5 provides detailed information on the clustering of these differential metabolites.

### Targeted metabolomics analysis of amino acid

Building upon our initial untargeted metabolomic analyses and corroborating findings from extant literature, we discerned that alongside lipid metabolites (such as SMs, PIs, and PCs), organic acids (such as acetic acid, taurine, N-undecanoylglycine), and nucleotides and analogues (such as inosine, pyruvic acid) identified in our preliminary untargeted examination, amino acid metabolism is intricately linked with OP. Despite the absence of significant variations in amino acids and their metabolites, aside from N-undecanoylglycine and phenylalanine, in our untargeted study, existing literature on the relationship between amino acid metabolism and OP (e.g., valine, lysine, threonine, methionine, tryptophan, and isoleucine) suggests a notable link [17, 18]. Consequently, we conducted targeted metabolomic validation of 37 amino acids and derivatives in cohort 2 plasma samples.

Through ANOVA analysis, we identified significant differences in six amino acids across the HC, OP, and ON groups (Fig. 3), specifically L-Aspartic acid (Asp), L-Asparagine (Asn), L-Lysine (Lys), L-Threonine (Thr), L-Citrulline (Cit), and Ethanolamine (EtN). Compared to the HC group, concentrations of Asp, Asn, Lys, Thr, and EtN were significantly reduced in the OP group, while Cit concentration was significantly increased. Notably, Asp, Asn, Cit, and EtN exhibited significant changes during the ON stage. Furthermore, the concentrations of Asn and Lys showed a positive correlation with BMD, in contrast to Cit, which exhibited a negative correlation with BMD. The quantification results for these six amino acids are presented in Table 3.

**Fig. 4:**
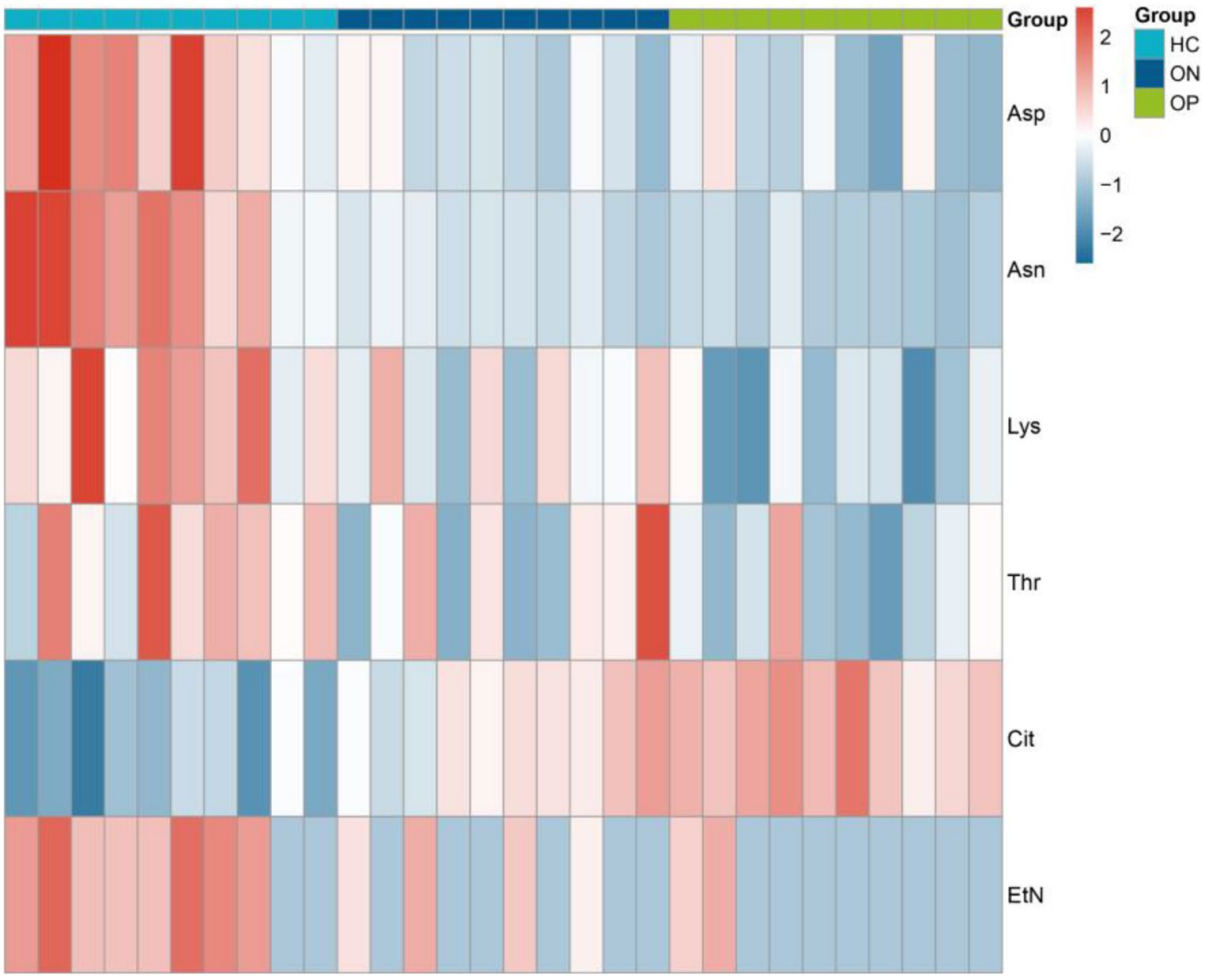
Differential amino acid levels in targeted metabolomics results. Asp, L-Aspartic acid; Asn, L-Asparagine; Lys, L-Lysine; Thr, L-Threonine; Cit, L-Citrulline; EtN, Ethanolamine

**Table 3:** The mean values of concentrations (µg/mL) of six differential amino acid in this study

Metabolite	HC	Patients (ON + OP)	ON	OP
L-Aspartic acid	0.25 ± 0.07	0.14 ± 0.03	0.15 ± 0.03	0.14 ± 0.04
L-Asparagine	0.76 ± 0.25	0.26 ± 0.07	0.3 ± 0.06	0.22 ± 0.06
L-Lysine	553 ± 44.09	487.3 ± 40.99	507.9 ± 36.31	466.7 ± 35.87
L-Threonine	12.21 ± 1.79	10.41 ± 1.97	10.86 ± 2.31	9.96 ± 1.55
L-Citrulline	3.29 ± 0.62	5.04 ± 0.59	4.71 ± 0.54	5.38 ± 0.45
Ethanolamine	0.15 ± 0.08	0.05 ± 0.06	0.06 ± 0.06	0.04 ± 0.06

Notes All data were presented as mean ± SD

**Fig. 5:**
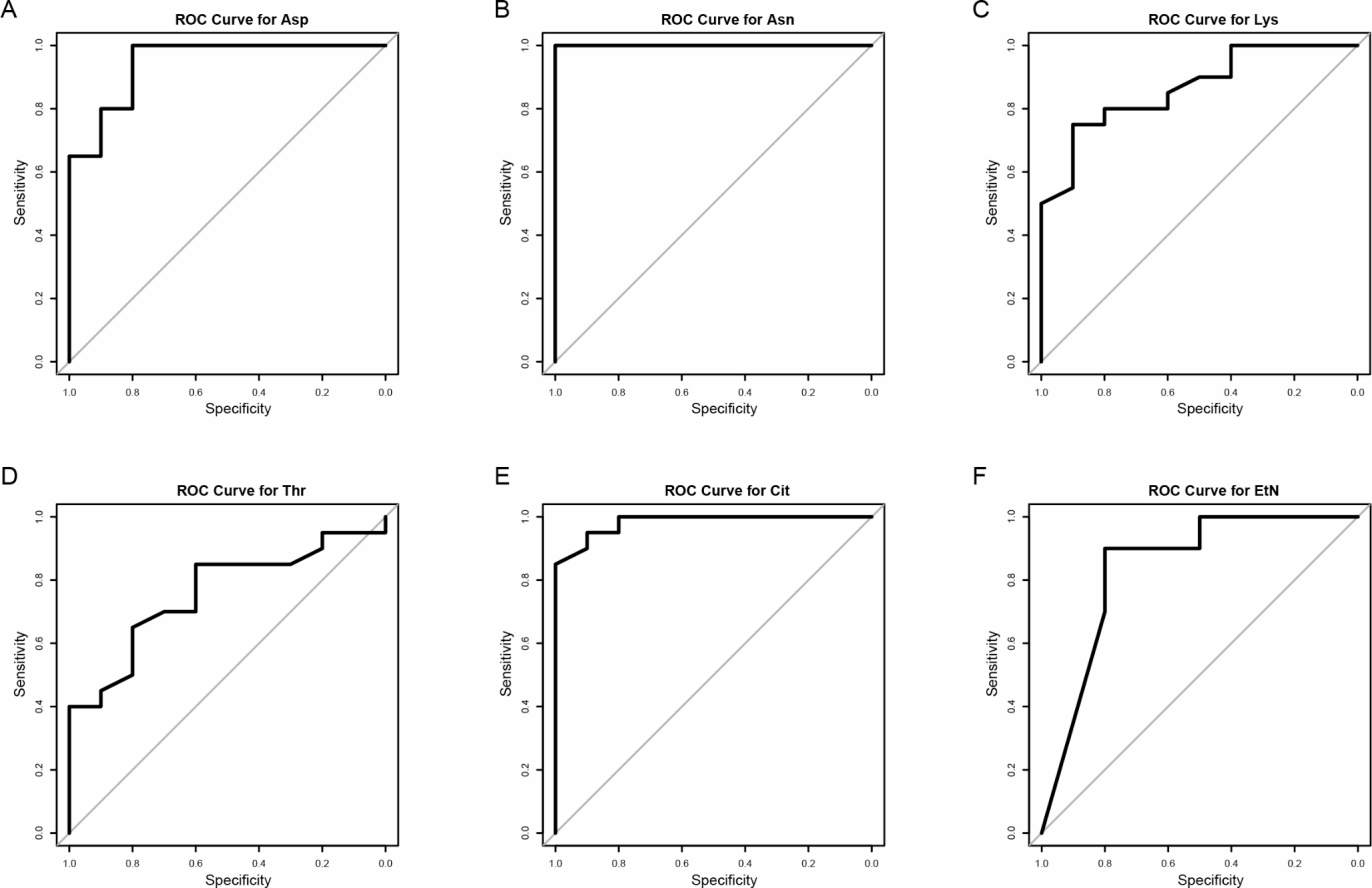
ROC curves for the six amino acids selected in the targeted analysis. Asp, L-Aspartic acid; Asn, L-Asparagine; Lys, L-Lysine; Thr, L-Threonine; Cit, L-Citrulline; EtN, Ethanolamine

Subsequently, we performed ROC curve analysis on the aforementioned six amino acids. The ROC curves for amino acids are depicted in Fig. 5, with specific data presented in Table 4. The amino acid with the highest diagnostic capability was Asn (AUC = 1.000), followed by Cit and Asp (AUCs of 0.983 and 0.945, respectively). The AUCs for Lys, EtN, and Thr were 0.865, 0.840, and 0.758, respectively.

**Table 4:** ROC curve analysis for the molecules selected in the targeted metabolomic profiling (HC vs.ON + OP)

Metabolite	Molecular Weight	AUC	95%CI	*P*-value
L-Aspartic acid	133.1	0.945	0.862–0.945	< 0.001
L-Asparagine	132.12	1.000	1.000–1.000	0
L-Lysine	146.19	0.865	0.734–0.865	< 0.001
L-Threonine	119.12	0.758	0.580–0.758	0.004
L-Citrulline	175.19	0.983	0.949–0.983	< 0.001
Ethanolamine	61.08	0.840	0.669–0.840	< 0.001

## Discussion

According to data from the International Osteoporosis Foundation, approximately one-third of women over the age of 50 worldwide will experience a fracture due to OP. Following a fracture, about 80% of patients remain undiagnosed with OP and do not receive treatment [19]. Thus, developing diagnostic tools capable of early diagnosis and differentiation between ON and OP is of significant importance. Our study focused on the primary patient population for ON and OP (postmenopausal women). We conducted the untargeted metabolomic analysis and the targeted metabolomics analysis of amino acid on patients diagnosed with ON and OP at the Third People’s Hospital of Mianyang City, comparing with a healthy control group. We aimed to explore differential metabolites using LC-MS to further discover potential biomarkers for early diagnosis. In our untargeted metabolomic analysis, we observed alterations in lipid and organic acid levels in the plasma of patients with ON and OP compared to the HC group. Notably, concentrations of metabolites such as acetic acid, taurine, inosine, pyruvic acid, and N-undecanoylglycine were found to correlate positively or negatively with disease progression. In the targeted metabolomics analysis of amino acid, we quantitatively assessed 37 amino acids and their derivatives. This analysis revealed significant variations in six specific amino acids (aspartic acid, asparagine, lysine, threonine, citrulline, and ethanolamine) in patients with ON and OP compared to the HC group.

Both cohort 1 and cohort 2 exhibited significant differences in BMD, a commonly used diagnostic measure for bone health. Clinical baseline analyses revealed no statistically significant differences in sample size, body mass index (BMI), fasting blood glucose (FBG), cholesterol, triglycerides (TG), high-density lipoprotein (HDL), or low-density lipoprotein (LDL) among the groups in both cohorts. However, in cohort 1, both the ON and OP groups exhibited lower t-scores. In cohort 2, both the ON and OP groups were significantly older (OP: 66.7 years > ON: 60.4 years > HC: 54.4 years) and also showed lower t-scores. Data from the Johns Hopkins Arthritis Center indicate that in postmenopausal Caucasian women in the United States, the prevalence of OP is 14% among those aged 50–59, 22% among those aged 60–69, 39% among those aged 70–79, and as high as 70% among those aged 80 and above [20]. This underscores the significantly increased risk of OP with advancing age, particularly among women. It is important to note that age is a significant confounding factor that may influence metabolite concentrations and biomarker expression. If not adequately controlled, age-related variations could lead to misinterpretations of metabolite differences, thereby affecting the validity of the results. Future analyses should consider adjusting for age to clarify its impact on the observed metabolic profiles.

Untargeted metabolomics revealed abnormalities in lipid metabolism in ON and OP patients. Several SMs and PIs correlated positively with BMD, while various PCs showed positive/negative correlations with BMD, which were similar to the findings of some previous studies [15, 21]. SMs, as principal components of double membrane-bound sphingolipids, are not only related to inflammation, cell survival, and proliferation but also play a significant role in bone formation and normal mineralization [22]. Abnormalities in SM metabolism can lead to bone mineralization defects, including OP, neonatal fractures, and osteogenesis imperfecta [23]. Mutations in the Sphingomyelin Synthase 2 gene (SGMS2) have been associated with potential bone mineralization defects. Pekkinen et al. conducted next-generation sequencing on six patients with OP and rare skeletal phenotypes, identifying SGMS2 mutations, underscoring the critical role of SM metabolism in bone health [24]. PIs play a crucial role in the PI3K/Akt/FoxO signaling pathway. Activation of cell surface receptors recruits and activates Phosphoinositide 3-kinase (PI3K), which phosphorylates phosphatidylinositol-4,5-bisphosphate (PIP2) to phosphatidylinositol-3,4,5-trisphosphate (PIP3), further activating protein kinase B (Akt) and regulating downstream Forkhead box O (FoxO) to modulate bone formation and resorption [25]. PC (18:3/18:3), PC (18:2/18:2), PC (18:3/20:4), PC (22:/22:5) are positively correlated with BMD, while PC (16:0/18:1), PC (18:0/18:2) are negatively correlated with BMD. Similar to our results, previous studies also found PCs to be associated with BMD in varying directions [26, 27]. Integrating these results suggests a close relationship between lipid metabolism and the pathogenesis of ON/OP. Thus, further lipidomic analyses are needed to delve deeper into the lipid changes associated with ON/OP.

We also discovered that the concentrations of taurine, inosine, pyruvic acid, and acetic acid increased in cases of OP. Research by Panahi and colleagues found that serum taurine levels were higher in healthy volunteers compared to those with ON/OP [16]. Taurine has been considered a potential therapeutic candidate for preventing postmenopausal OP due to its ability to improve alkaline phosphatase (ALP) activity and mineralization and inhibit osteoclast formation during bone resorption [28]. Uric acid, known for its antioxidant capacity in preventing the formation of peroxynitrite, may help prevent osteoporosis [29]. Previous studies have also shown that serum uric acid levels are negatively correlated with decreased BMD and increased fracture risk [30, 31]. Inosine, a precursor to uric acid, was first reported by Mei et al. to be negatively associated with low BMD states [27]. It is worth noting that the changing trends of taurine and inosine in our study were opposite to those of these studies. This discrepancy could be due to compensatory responses or exogenous intake, though unfortunately, we cannot provide an accurate explanation. Pyruvic acid serves as a crucial intermediate linking glycolysis and the tricarboxylic acid (TCA) cycle, while acetic acid acts as a form in which it participates in the TCA cycle via acetyl coenzyme A. When OP occurs, it may influence the metabolic state of the organism. Any factors that affect the entry of pyruvic acid into mitochondria for the synthesis of acetyl coenzyme A could potentially lead to the accumulation of pyruvic acid and acetic acid in the bloodstream [32].

Our untargeted metabolomics analysis only identified two different amino acids and derivatives, N-undecanoylglycine and phenylalanine, despite amino acids and derivatives often being associated with decreased BMD in the literature [33]. In this context, we conducted targeted metabolomic validation of 37 amino acids and derivatives in cohort 2 plasma samples and identified six closely related to osteoporosis. A systematic review on metabolites associated with human BMD suggested that glycine was negatively correlated with BMD, valine positively correlated, while the relationship between leucine/isoleucine and BMD was inconsistent [13]. Other studies have also suggested potential associations between BMD and amino acids including threonine, aspartic acid, citrulline, and alanine [21, 34, 35]. In vitro studies have confirmed that essential amino acids (threonine, methionine, tryptophan, arginine) can regulate osteoblast growth and differentiation [36]. Notably, our investigation revealed that N-undecanoylglycine was significantly downregulated during the osteopenic phase, with an even more pronounced decrease in the osteoporotic stage. The production of N-undecanoylglycine relies on the availability of undecanoic acid and glycine, as well as the reaction rate between these components, which may be affected in the context of osteoporosis. However, we could not establish direct evidence regarding the specific role of N-undecanoylglycine and its metabolic changes in osteoporosis. It is important to note that our study has limitations, particularly in cohort 2, where each group comprised only 10 participants. Additionally, the significant age differences among the groups may confound the results, potentially influencing the observed metabolite differences. These factors highlight the need for further research to elucidate the mechanisms by which these amino acids and derivatives affect osteoporosis.

Over the past decade, metabolomics has increasingly been applied in the identification of disease biomarkers, now considered a tool with potential for clinical translation [37]. However, the clinical translation and widespread implementation of metabolomics still face several challenges. Firstly, taking OP as an example, while certain metabolites are indeed associated with OP, it remains to be further determined whether these differential metabolites play a direct causal role in the pathogenesis of OP or are the result of other diseases and confounding factors [38, 39]. Furthermore, standardization in clinical-grade laboratories is crucial, necessitating uniform standardization of reporting and data analysis to ensure the comparability and credibility of results. Absolute quantification across different platforms is vital, yet many studies have not achieved this standard. Lastly, the analysis of metabolomics results requires the collaboration of multidisciplinary professionals to meet the demands of high-end instrument platforms and specialized data processing algorithms [40].

Our untargeted metabolomics analysis identified several lipid metabolites (e.g., SMs, PIs, and PCs), organic acids (e.g., acetate, taurine, N-undecanoylglycine), and nucleotides and analogues (e.g., inosine, pyruvate) as the most significant differential metabolites among the three groups. Correlation analysis indicated that N-undecanoylglycine, short peptides, and phosphatidylinositol were positively correlated with BMD, whereas acetic acid, phenylalanine, taurine, inosine, and pyruvate showed negative correlations with BMD. Additionally, c-means clustering analysis revealed that uric acid and lysophosphatidic acid were significantly enriched during the osteonecrosis stage, suggesting their potential as early diagnostic markers for osteonecrosis. In the amino acid levels, we observed significant differences only in N-undecanoylglycine and phenylalanine. However, it is important to acknowledge several limitations in our study: (1) Selective Focus on Amino Acids: This project primarily concentrated on amino acids, performing targeted metabolomics analysis without exploring other major differential metabolites identified in the untargeted analysis, such as lipid metabolites and organic acids. This selective approach may limit our understanding of the overall metabolic changes and overlook the potential roles of other important biomarkers. (2) Limited Findings from Cohort 1: In the untargeted metabolomics analysis of cohort 1 (*n* = 96), only N-undecanoylglycine and phenylalanine were found to differ significantly. This limited scope may not fully capture the complexity of the metabolic networks associated with changes in BMD. Furthermore, cohort 2 was subsequently used for targeted metabolomics analysis focused solely on amino acids, neglecting further exploration of other significant metabolites identified in the untargeted analysis. (3) Small Sample Size of Cohort 2: With only 30 cases in cohort 2, the statistical power of our analyses may be limited, potentially compromising the reliability and generalizability of the results. Small sample sizes increase the risk of false negatives or false positives, which can hinder a comprehensive understanding of the targeted metabolomics analysis of amino acids. (4) Age as a Confounding Factor: Age is a known risk factor for osteonecrosis and osteoporosis. The significant age differences within cohort 2 were not controlled for, which may influence metabolite concentrations and biomarker expression, further complicating the interpretation of our results. (5) Lack of Supporting Literature: Although we identified several differential metabolites, there is insufficient scientific background to explain the inconsistencies between our findings and existing studies. Future investigations should consider larger sample sizes and explore potential confounding factors that may affect plasma metabolite profiles.

## Conclusion

Our untargeted metabolomics analysis identified several differential metabolites associated with BMD, including various lipid metabolites, organic acids, and nucleotides, with significant differences observed in N-undecylglycine and phenylalanine levels. Limitations of this study include a selective focus on amino acids, a small sample size in cohort 2, and unaddressed age differences, which may hinder a comprehensive understanding of metabolic changes in osteoporosis. Future research should expand sample sizes and consider additional confounding variables to clarify the roles of these metabolites. Our findings offer valuable insights into the early diagnosis and etiology of osteoporosis, potentially guiding the development of new diagnostic methods and therapeutic strategies.

## Electronic supplementary material

Below is the link to the electronic supplementary material.

Supplementary Material 1

Supplementary Material 2

## Data Availability

No datasets were generated or analysed during the current study.
