# Supplementary figures and images for: Combining untargeted and targeted metabolomic profiling reveals principal differences between osteopenia, Osteoporosis and healthy controls
Source: Aging Clin Exp Res. 2025 Jan 21;37(1):28. doi: 10.1007/s40520-024-02923-3 (PMC11746959; doi:10.1007/s40520-024-02923-3)

**Supplementary figure 1**


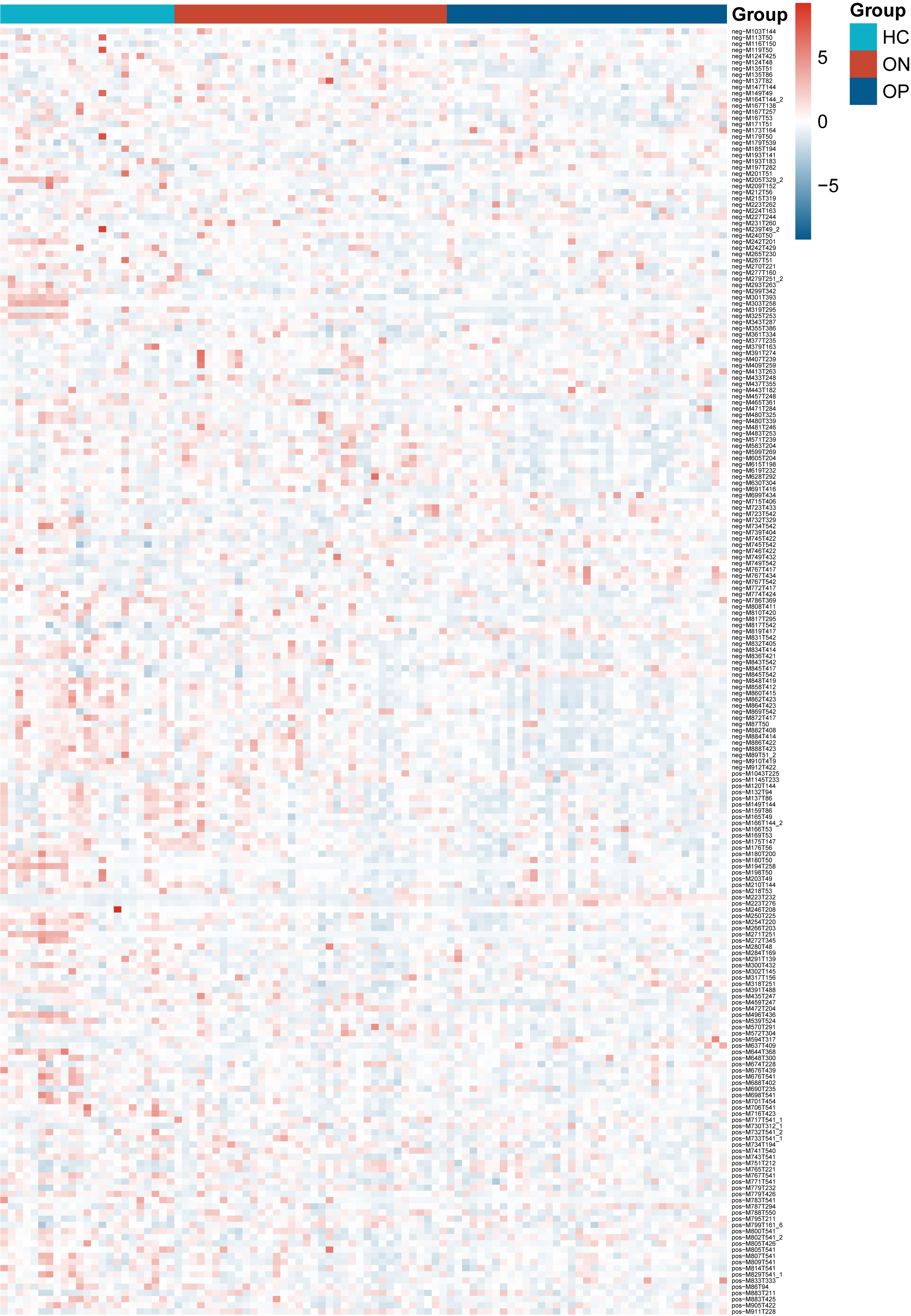


**Supplementary figure 2**


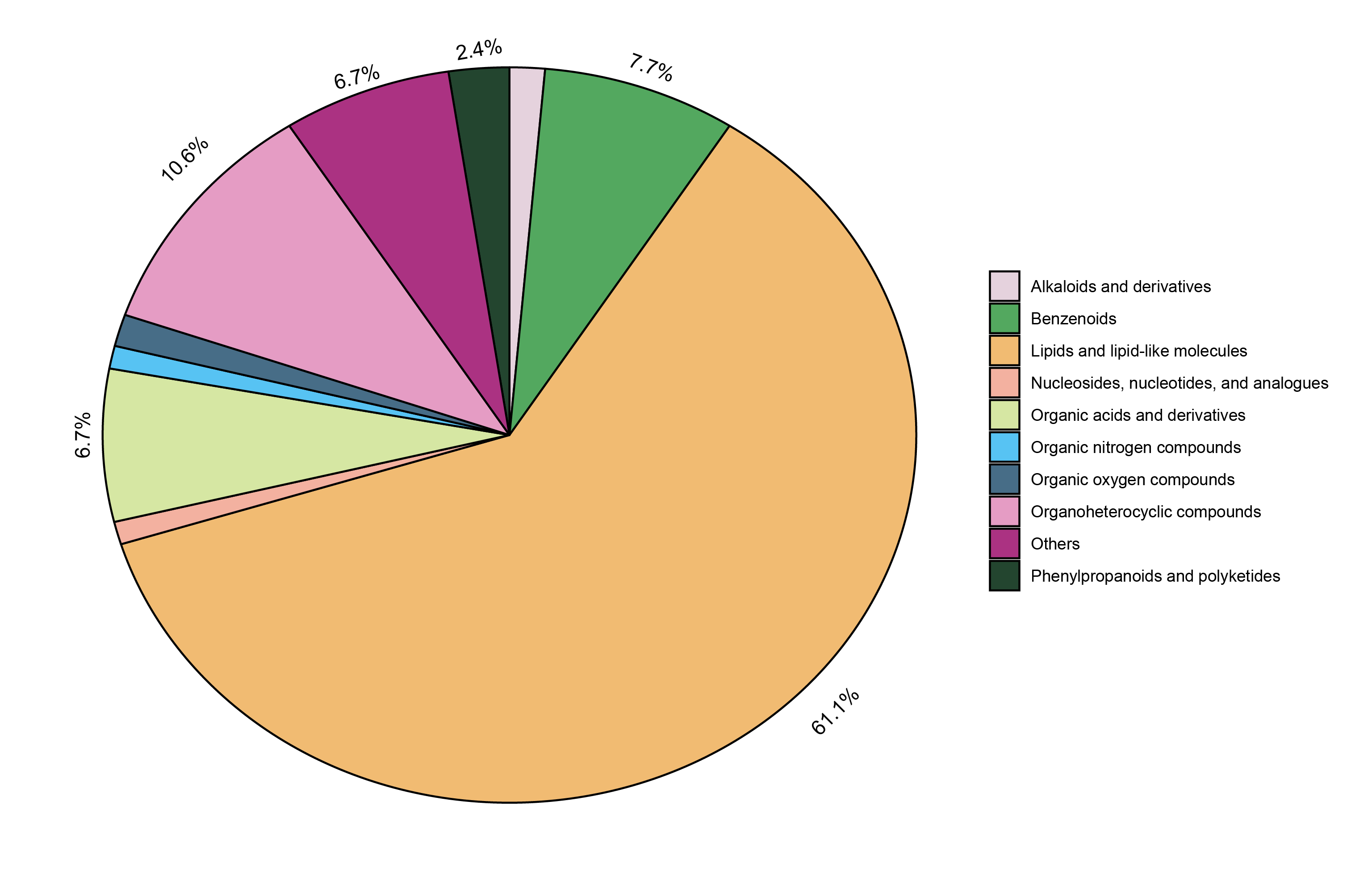

Supplement: Supplementary file 1 — Supplementary Material 1 [file 40520_2024_2923_MOESM1_ESM.docx]
